# Evaluation of the clip anchorage technique using mucosal elevation and incision in prevention of esophageal stent migration

**DOI:** 10.1055/a-2663-6533

**Published:** 2025-08-19

**Authors:** Anais Darnaude, Maxime Thobois, Philippe Onana Ndong, Cécile Gomercic, James Boulant, Geoffroy Vanbiervliet

**Affiliations:** 1 37045 Gastroenterology, CHU Nice, Nice, France; 2 Gastroenterology, Clinique du Palais, Grasse, France

**Keywords:** Endoscopy Upper GI Tract, Benign strictures, Malignant strictures, Dilation, injection, stenting

## Abstract

**Background and study aims:**

Migration remains a frequent and challenging situation following esophageal stent placement. The aims of this study were to evaluate efficiency and safety of a new anchorage technique using through-the-scope (TTS) clips to prevent esophageal stent migration.

**Patients and methods:**

This was a retrospective case-control analysis of a prospective, monocentric database. Patients with a fully-covered esophageal stent, fixed or not for benign or malignant indications, were included. Fixation of the stent at the oral flange was achieved with TTS clips, placed for a bite in submucosal space after injection with saline and mucosal incision using the tip of a snare.

**Results:**

A total of 52 patients were included, 24 stents with anchorage (fixed group) and 28 without (control group). Fixation was more frequently performed for benign disease (75.0% fixed group vs. 39.29% for control, *P* = 0.021). Median length of stent dwell time was 41.5 days in the fixed group and 30.5 days for controls ( *P* = 0.263). The overall migration rate was comparable (45.83% in the fixed group vs. 35.71% for controls, *P* = 0.647). A higher rate of early migration was observed in the control group (60.0% vs 18.18%, *P* = 0.080). A prior history of radio-chemotherapy was predictive of migration. There was no increased complication rate at placement or at removal in the treated group.

**Conclusions:**

The new esophageal stent fixation technique appears to be simple, inexpensive, feasible, and safe. Although there is no impact on overall migration, there does seem to be a reduction in early migration.

## Introduction

Esophageal stenting is an effective and minimally invasive procedure that has been validated by several scientific endoscopic societies for management of both benign and malignant diseases of the esophagus 1 . Historically, esophageal stents have been employed for management of dysphagia in the context of inoperable cancer. Advancement of materials has facilitated gradual expansion of indications for stenting 2 3 . The initial rigid plastic stents were rapidly superseded by uncovered metallic self-expanding esophageal stents (SEMS) due to their reduced complication and migration rates 4 . Nevertheless, use of these uncovered SEMS was associated with obstructions due to ingrowth tumoral expansion through the mesh and/or overgrowth hyperplastic and granular tissue at the tip of the stent 5 . Subsequently, a new generation of covered esophageal stents was developed, incorporating a silicone or polyurethane membrane covering the mesh. These had the advantage of being removable, thereby enabling their use to be extended to benign pathologies such as anastomotic stricture, fistulas and perforations 6 7 .

Despite technical advancements, stent migration remains the most prevalent complication, especially when a fully covered self-expandable metal stent (FCSEMS) is used in the context of benign pathology 8 9 10 . Several techniques have been described to fix the stent and limit the phenomena. Vanbiervliet et al. demonstrated that anchoring the FCSEMS to the esophageal mucosa using hemostatic clips resulted in a reduced rate of migration 11 . In 2020, Singla et al. described a new technique using a through-the-scope (TTS) clip after mucosal incision to improve anchorage to the esophageal mucosa 12 . To the best of our knowledge, no studies have evaluated efficacy of this anchoring technique.

The main objective of this study was to assess efficacy of FCSEMS fixation by TTS clip following mucosal incision in prevention of migration. Secondary objectives were to determine the safety of the technique, factors associated with migration, and time to migration after stent placement.

## Patients and methods

### Patients

We conducted a single-center retrospective comparative analysis of data prospectively collected from patients aged ≥ 18 years who underwent FCSEMS placement for benign or malignant esophageal disease at the University Hospital of Nice in France between November 1, 2020 and the April 30, 2024. Patients were excluded from the analysis if the esophageal stent was anchored using a technique other than that studied. Written consent was obtained from the entire study population after they had been informed about the procedure and its complications. Each patient gave their consent for use of their per protocol data. The study protocol conforms to the ethical guidelines of the 1975 Declaration of Helsinki.

The primary endpoint was FCSEMS migration as defined below. Secondary endpoints were occurrence of adverse events (AEs) associated with the procedure or during the follow-up and the time between stent placement and migration.

### Endoscopic technique

#### Stent placement

The endoscopic esophageal stenting technique was performed in accordance with the established standard 13 , under fluoroscopic control (OEC 9900, GE Healthcare, Little Chalfont, United Kingdom) throughout. An initial opacification in the supine position was employed to evaluate the anatomy, including presence and characteristics of any stricture, fistula, and the location, length, and caliber of the affected segment. Subsequently, the stricture was catheterized under fluoroscopic control with a stiff guide wire. The stent could then be inserted either in parallel to the gastroscope (over-the-wire or OTW technique) or through the working channel of the endoscope (TTS technique), without prior dilation. The stents used were Wallflex (Boston Scientific, Marlborough, Massachusetts, United States), Niti-S (Taewoong Medical, Gyeonggi-do, South Korea), and Hanaro (M.I.Tech, Gyeonggi-do, South Korea). Dimensions of the stents were selected in accordance with the height and position of the stricture to maintain a minimum distance of 2 cm above the upper and lower poles of the obstruction or fistula as recommended 1 .

#### Stent fixation

**Fig. 1:**
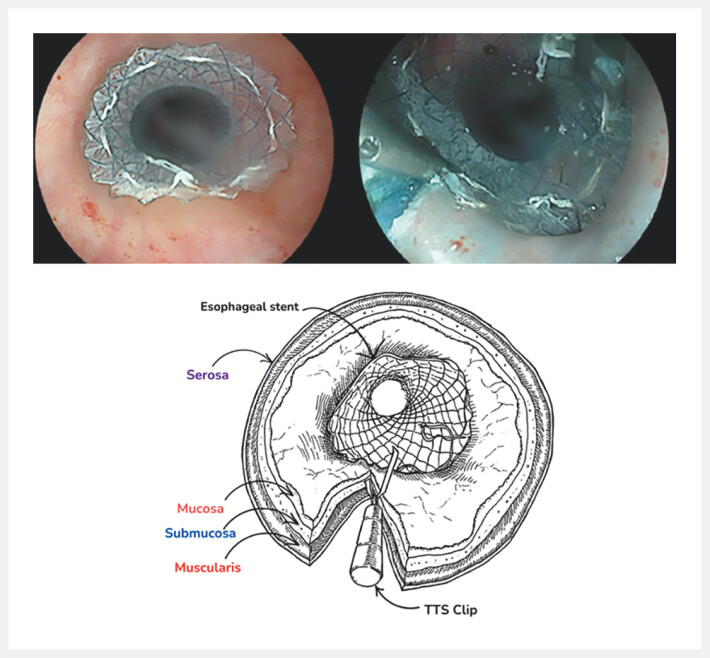
Stent fixation.

Patients were included successively over the study period, initially without fixation and then with fixation. Fixation of the stent was first performed in the conventional way, using a TTS clip, and then using the technique described above in a submucosal position ( Fig. 1 ). Following placement of the esophageal stent, the mucosa was lifted using a 25G injection needle (Interject, Boston Scientific, Marlborough, Massachusetts, United States) with indigo carmine saline. Subsequently, a mucosal incision was made using the tip of a rigid 10-mm monofilament snare (Fujifilm Medwork, Hochstadt/Aisch, Germany) with an electrical generator (VIO3, Erbe Elektromedizin, Tübingen, Germany) in dissection mode (Endocut I Effect 2 - Section width 2 - Section interval 2). Subsequently, a clip (Resolution, Boston Scientific, Marlborough, Massachusetts, United States) was inserted using a TTS technique to secure the upper flange of the esophageal stent. One edge of the clip was inserted into the submucosal space, while the other edge gripped the stent. A second clip was placed using the same procedure in the majority of cases on the opposite side of the digestive lumen. Only one stent had been fixed with a single TTS clip. In the present study, the aforementioned technique was performed by three expert endoscopists (CG, JB, GV), who conducted a review of the fixation technique employed during the first case.

### Migration

Migration was defined as presence of endoscopic or radiological evidence of dislodgement of the stent, occurring either upstream or downstream of the area initially treated (as observed in the final fluoroscopic image obtained during the initial endoscopic procedure). Classification of esophageal stent migration was based on the time frame in question. The term "early migration" was used to describe events occurring within 30 days of stent insertion, whereas "late migration" was used to describe events occurring after this period.

### Patient follow-up

The end date of follow-up was determined based on the date of device removal, the date of stent migration discovery, or the date of patient death. In the event of patient mortality during the follow-up period, the esophageal stent was deemed to remain in situ absent clinical indications suggestive of migration or absent endoscopic or radiological evidence substantiating it.

Complications that occurred during insertion of the stent, the insertion period or removal were classified according to the AGREE classification (International Classification for Adverse Events in Gastrointestinal Endoscopy) 14 .

### Statistics

Numerical variables were expressed as mean (±SD). Time variables were expressed as median (interquartile range). Discrete results were expressed as absolute and relative frequencies (%). We compared two groups: the fixed group and the control group. Group comparability was assessed by comparing baseline demographic data and follow-up duration between groups. Normality and heteroskedasticity of continuous data were assessed with Shapiro-Wilk and Levene’s test respectively. Continuous outcomes were compared with unpaired Student *t* -test, Welch t-test or Mann-Whitney U test according to data distribution. Discrete outcomes were compared with chi-squared or Fisher’s exact test accordingly. The alpha risk was set to 5% and two-tailed tests were used. Statistical analysis was performed with EasyMedStat (version 3.36).

## Results

### Patient characteristics

**Fig. 2:**
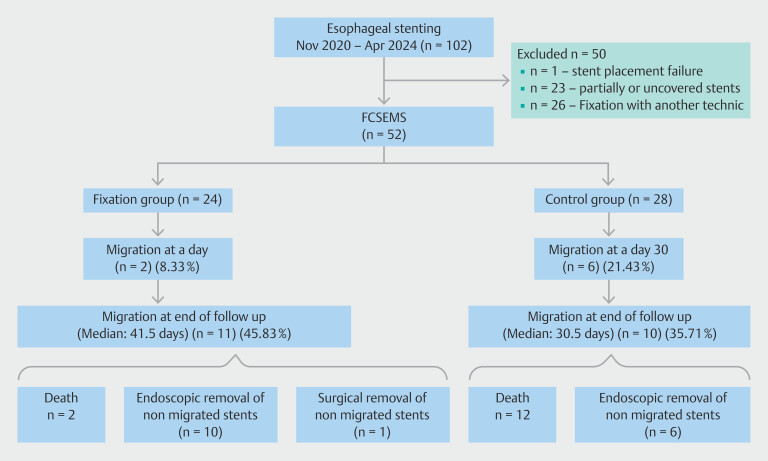
Flowchart.

Of the 102 patients who underwent esophageal stenting, 50 were excluded from the analysis (one stent placement failure, 23 partially covered or uncovered stents, and 26 stents fixed with TTS clips without mucosal incision). The flow chart is shown in Fig. 2 . A total of 52 patients were included in the study, comprising 24 in the fixed group and 28 in the control group. A summary of patient characteristics is provided in Table 1 . In 55.8% of cases (n = 29/52), the FCSEMS was placed for a benign etiology. Stricture constituted half of the indications, the majority of which were tumor-related (51.3%, n = 20/52).

**Table:** **Table 1** Baseline patient characteristics.

	**Overall n = 52**	**Fixed group n = 24**	**Control group n = 28**	**P value**
**Age** , mean, years ± SD [95%CI]	62.9 ± 12.95 [59.35–66.45]	60.88 ± 13.58 [55.14–66.61]	64.64 ± 12.63 [59.75–69.54]	0.305
**Gender** , male n (%)	34 (65.4)	12 (50.0)	22 (78.57)	**0.043**
ASA score, n (%)				0.126
ASA 1	1 (1.9)	-	1 (3.57)	
ASA 2	18 (34.6)	12 (50.0)	6 (21.43)	
ASA 3	28 (53.8)	10 (41.67)	18 (64.29)	
ASA 4	5 (9.6)	2 (8.33)	3 (10.71)	
**Benign/malignant pathology** , n (%)				**0.021**
Benign	29 (55.8)	18 (75.0)	11 (39.29)	
Malignant	23 (44.2)	6 (25.0)	17 (60.71)	
**Stenosis – fistula/perforation** , n (%)				0.230
Stricture	26 (50.0)	9 (37.5)	17 (60.71)	
Fistula or perforation	13 (25.0)	8 (33.33)	5 (17.86)	
Stricture with fistula or perforation	13 (25.0)	7 (29.17)	6 (21.43)	
**Type of stricture** , n (%)	n = 39	n = 16	n = 23	0.200
Malignant	20 (51.3)	6 (37.5)	14 (60.87)	
Benign	19 (48.7)	10 (62.5)	9 (39.13)	
**Non-crossable stricture** , n (%)	29/39 (74.4)	9/16 (56.25)	20/23 (86.96)	0.060
**Location** , n (%)				**0.011**
Anastomosis	14 (26.9)	9 (37.5)	5 (17.86)	
Upper	9 (17.3)	-	9 (32.14)	
Middle	7 (13.5)	4 (16.67)	3 (10.71)	
Lower	22 (42.3)	11 (45.83)	11 (39.29)	
**History of surgery** , n (%)	n = 21	n = 13	n = 8	0.410
Oncological surgery	12 (57.1)	7 (53.85)	5 (62.5)	
Bariatric surgery	6 (28.6)	5 (38.46)	1 (12.5)	
Isotactic junction surgery	3 (14.3)	1 (7.69)	2 (25.0)	
**Prior oncological therapy** , n (%)*	n = 27 (52.9)	11/24 (45.83)	16/27 (59.26)	0.498
Radiotherapy	3 (5.9)	1 (4.17)	2 (7.41)	> 0.999
Radio-chemotherapy	5 (9.8)	2 (8.33)	3 (11.11)	-
Chemotherapy	18 (35.3)	8 (33.33)	10 (37.04)	> 0.999
Immunotherapy	7 (13.7)	3 (12.5)	4 (14.81)	> 0.999
Hormonotherapy	1 (2.0)	-	1 (3.7)	> 0.999
**Concomitant oncological therapy** , n (%)*	7 (13.7)	2/24 (8.33)	5/27 (18.52)	0.425
Radiotherapy	1 (2.0)	-	1 (3.7)	> 0.999
Radio-chemotherapy	-	-	-	-
Chemotherapy	4 (7.8)	2 (8.33)	2 (7.41)	> 0.999
Immunotherapy	3 (5.9)	1 (4.17)	2 (7.41)	> 0.999
Hormonotherapy	1 (2.0)	-	1 (3.7)	> 0.999
Priori endoscopic dilation, n (%)	14 (26.92)	9 (37.5)	5 (17.86)	0.130
*Analysis of 51 patients: One missing data. ASA, American Society of Anesthesiologists; CI, confidence interval; ESD, endoscopic submucosal dissection; SD, standard deviation				

The control group was composed of a greater proportion of male patients (n = 22/28; 78.57% vs. n = 12/24; 50%). With regard to overall indications (stricture and wall defect), the fixed group exhibited a significantly higher prevalence of benign pathology (n = 18/24; 75% vs. n = 11/28; 39.29%, *P* = 0.021) and higher incidence of anastomotic stricture covered than the control group (n =9/24; 37.5% vs. n = 5/28; 17.86%, *P* = 0.011).

### Characteristics of stents and follow-up

Median duration of stent therapy was comparable between the two groups, as were the size and caliber of the FCSEMS ( Table 2 ). Median stenting time was found to be significantly longer in the fixed group compared with the control group (26.5 vs. 19.5 minutes, *P* = 0.006). During the follow-up period, the mortality rate was significantly higher in the control group (n = 12/28; 42.86% vs. n = 2/24; 8.33%), *P* = 0.011).

**Table:** **Table 2** Stenting characteristics and follow up.

	**Fixed group n = 24**	**Control group n = 28**	***P* value **
**Mean diameter of stent** , mm ± SD [95%CI]	21.12 ± 3.19 (19.78–22.47)	19.79 ± 3.62 (18.38–21.19)	0.121
**Mean length of stent** , mm ± SD [95%CI]	95.83 ± 28.88 (83.64–108.03)	94.11 ± 28.32 (83.13–105.09)	0.836
**Median duration for stent placement** , min (IQR)	26.5 (21.75–35)	19.5 (17–27)	**0.006**
**Median time of stent therapy** , days (IQR)	41.5 (16.5–85.75)	30.5 (12.5–51.25)	0.263
**Death during follow-up** , n (%)	2 (8.33)	12 (42.86)	**0.011**
CI, confidence interval; IQR, interquartile range; SD, standard deviation.			

### Stent migration

**Fig. 3:**
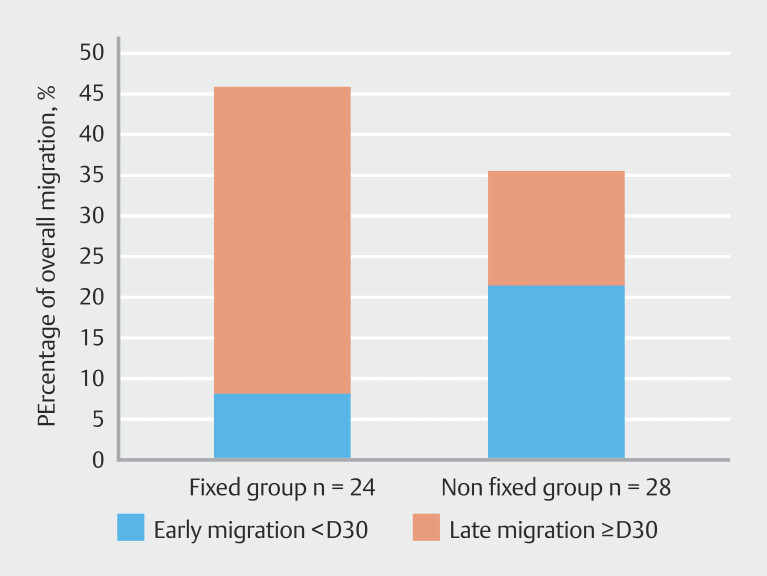
Proportion of stent migration depending on fixation.

The overall migration rate was comparable in both groups (n =11/24; 45.83% in the fixed group vs. n = 10/28; 35.71%) in the control group, *P* = 0.6 47) ( Table 3 , Fig. 3 ). Nevertheless, there was a tendency for the control group to exhibit a higher early migration rate (n = 6/18; 60.0% vs. n = 2/24; 18.18%, *P* = 0.080). Among the 13 patients whose fixed stent had not migrated, seven had the clips still in place at the time of stent removal, whereas one patient had the stent still in place without the clips. The remaining five patient cases lack this information.

**Table:** **Table 3** Stent migration.

	**Fixed group n = 24**	**Control group n = 28**	***P* value **
Overall migration, n (%)	11 (45.83)	10 (35.71)	0.647
Time to migration	n = 11	n = 10	0.08
Early migration, n (%)	2 (18.18)	6 (60.0)	
Late migration, n (%)	9 (81.82)	4 (40.0)	

### Factors associated with migration

A history of radio-chemotherapy was identified as a significant predictor of stent migration. Among patients whose stent had migrated, 23.81% (n = 5/21) had received this type of treatment prior to stent placement. Conversely, none of the patients whose stent remained in place had received such treatment ( *P* = 0.009). Esophageal dilation prior to stent implantation also appears to be a factor influencing risk of migration. Among patients with migration, 42.86% (n = 9/21) had undergone prior dilation, compared with 16.13% (n = 5/31) in the group of patients whose stent did not migrate ( *P* = 0.055). Characteristics of FCSEMS and median duration of stent therapy were not found to be associated with risk of migration.

### Removability

Of the 13 fixed non migrated stents, 10 were removed without incident. Of the three non-removed stent, two concerned patients who died during follow up, and the last was removed during a surgery.

### Safety

Twenty complications were reported during the follow-up (overall rate of 38.4%). None were observed during stent insertion or removal in either group. Incidence of complications during stent therapy was slightly higher in the fixed group but not statically significant (n = 12; 50.0% vs. n = 8; 28.57%, *P* = 0.052). Nevertheless, the majority of complications (n = 12) were of type I according to AGREE classification (esophageal ulcerations due to the flange of the stent, treated by proton pump inhibitors) and no difference between groups was observed when AGREE III or IV described (fixed group n = 3, 12% vs. control group n = 5, 18%). In the fixed group, a duodenal perforation resulting from esophageal stent migration constituted a grade IV complication. In the same group, two grade III complications were identified (stent intolerance due to an upstream and a downstream migration requiring endoscopic dilation and removal of the migrated material). In the control group, five grade III complications were identified. Three of the cases motivated early removal of the stent due to intolerance (pain or dyspnea caused by extrinsic compression of the trachea). The remaining two complications were formation of an esophageal bronchial fistula or fistulation of the stent to the skin.

## Discussion

Prevention of covered esophageal stent migration is still a challenge. In our study, we did not observe any advantage in using this new clip anchorage technique after incision of the mucosa regarding the overall migration rate. However, the rate of early migration seems to be reduced.

In 2012, Vanbiervliet et al. showed a reduction in migration rate of FCSEMS from 34% to 13% by fixing the proximal flange of the stent to the mucosa using hemostatic clips 11 . A randomized trial published in 2015 by Wang et al. subsequently confirmed the effectiveness of this technique in the context of tumor stricture 15 . Use of a modified technique with better grip of the esophageal mucosa by the clip thanks to its prior incision, as proposed by Singla et al. in 2020 12 , could have led to a superior effect in prevention of migration.

We did not observe this effect in our present study with an overall migration rate (40.4%) in line with the preexisting literature 16 17 . However, these negative results can be explained by various factors. First, the retrospective nature of the study led to a comparability bias between the treated and control groups. The rate of benign pathology was significantly higher in the fixed group than in the control group. However, Thomas et al. 18 showed that absent any fixation method, the rate of migration of completely covered esophageal stents in benign pathology was higher (30%) than in malignant stenosis (23%). These data were confirmed by a meta-analysis including 18 studies and 444 patients, which found an esophageal stent migration rate of 29% in cases of benign stenosis 10 . It, therefore, appears that the group of patients treated in our study had more risk factors for migration than the control group. Another factor that may explain our results is the high rate of death observed in the control group for a comparable length of follow-up in the two groups. This difference is probably linked to the greater representation of malignant pathologies in the control group. Absent evidence of migration, we considered the stent to be in place at the time of the patient's death. This difference in occurrence of death during follow-up between the two groups, therefore, may have reduced the rate of migration objectively observed in the control group. This bias could have been limited in a prospective study with regular radiological monitoring of stent position.

Nevertheless, this fixation technique could influence early migration. The difference in rate of early migration is not significant (18.18% (n = 2/24) in the fixed group vs. 60% (n = 6/18) the control group during the first month, *P* = 0.08), probably due to the small size of our population.

A later migration in the interventional group could easily be explained by healing of the incision and/or by stent movements and esophageal peristalsis. Furthermore, the significantly longer duration of the procedure when using this new technique is due to incision of the mucosa and placement of the clips, but appears to be safe.

Various alternative techniques have been developed to limit the migration rate of esophageal stents. Fixation using an over-the-scope clip (OTSC) was studied as early as 2014 with encouraging results. Mudumbi et al. showed a stent migration rate of 16.7% in the context of benign or malignant non-stenotic pathology but with an average follow-up of only 3 weeks 19 . A specific fixation device developed by OVESCO, the Stentfix OTS Clip System (OVESCO, AG, Tuebingen, Germany), was subsequently designed for this indication 20 . A single-center retrospective observational study published in 2022 by Schiemer et al showed a significant reduction in rate of migration from 35.4% to 8.3% with this system in the context of benign or malignant pathology, with a median follow-up time of 30 days 21 . Use of endoscopic sutures with OverStitch platform (Boston Scientific, Marlborough, Massachusetts, United States) has also been shown to be effective with a significant reduction in risk of migration from 63.4% to 19.0% including benign and malignant indications 22 . Finally, a meta-analysis published in 2023, which included 1,014 patients from 10 studies, demonstrated that stent fixation, regardless of the method employed, was associated with a notable reduction in rate of migration. That study revealed no significant difference in migration rates between the various techniques employed 23 . Consequently, although stent fixation has been shown to be an effective method for preventing stent migration on repeated occasions, the optimal technique remains undetermined. In the study by Mehta et al. 24 , the cost-effectiveness analysis indicated that the Stentfix OTS Clip System was the more cost-effective option. The OverStitch suture device is a costlier option than the Stentfix OTS Clip System. Furthermore, in the majority of cases, the Stentfix clip was removed concurrently with the stent using rat tooth forceps, whereas the OverStitch device necessitated utilization of endoscopic scissors, thereby augmenting the financial burden. In the present study, we did not include a cost-effectiveness analysis of stent anchorage using expected less expensive TTS clips.

Our work has several limitations. The retrospective nature of the study meant that the groups were not comparable, particularly in terms of distribution of benign and malignant pathologies. As we have shown, this may have contributed to the negativity of our results. A subsequent prospective study, with randomization stratified according to the benign or malignant aspect of the pathology and characteristics of the anatomical anomaly in question (stricture, leak, perforation, location of the area to be treated) would be the most relevant. Furthermore, the small number of patients did not allow statistical confirmation of the probably protective effect of the technique on early migration of completely covered material. However, this study reflects endoscopic practice in a “real-life”’ situation, confirming that this fixation method is safe, simple, and rapidly integrated and mastered.

## Conclusions

In conclusion, use of TTS clips for fixation of esophageal stents following submucosal injection and mucosal incision appears to be a viable and relatively low-risk procedure. Despite the technique failing to prevent migration in the present study, due to the biases previously demonstrated, it appears that migration may be delayed when this method is used. Furthermore, this technique is straightforward, cost-effective, and does not impair removability of the stent. These findings require confirmation in a prospective, randomized study with a larger number of patients, stratified according to pathology, with a concomitant cost-effectiveness analysis.
